# Efficient Achromatic Broadband Focusing and Polarization Manipulation of a Novel Designed Multifunctional Metasurface Zone Plate

**DOI:** 10.3390/nano11123436

**Published:** 2021-12-18

**Authors:** Shaobo Ge, Weiguo Liu, Xueping Sun, Jin Zhang, Pengfei Yang, Yingxue Xi, Shun Zhou, Yechuan Zhu, Xinxin Pu

**Affiliations:** Shaanxi Province Key Laboratory of Thin Films Technology and Optical Test, School of Optoelectronic Engineering, Xi’an Technological University, Xi’an 710032, China; geshaobo@126.com (S.G.); xuepingsun@xatu.edu.cn (X.S.); j.zhang@xatu.edu.cn (J.Z.); xiyingxue@163.com (Y.X.); zsemail@126.com (S.Z.); zyc_xatu@126.com (Y.Z.); pxx_1125@126.com (X.P.)

**Keywords:** metasurface, zone plate, achromatic

## Abstract

In this paper, comprehensively utilizing the diffraction theory and electromagnetic resonance effect is creatively employed to design a multifunctional metasurface zone plate (MMZP) and achieve the control of polarization states, while maintaining a broadband achromatic converging property in a near-IR region. The MMZP consists of several rings with fixed width and varying heights; each ring has a number of nanofins (usually called meta-atoms). The numerical simulation method is used to analyze the intensity distribution and polarization state of the emergent light, and the results show that the designed MMZP can realize the polarization manipulation while keeping the broadband in focus. For a specific design wavelength (0.7 μm), the incident light can be converted from left circularly polarized light to right circularly polarized light after passing through the MMZP, and the focusing efficiency reaches above 35%, which is more than twice as much as reported in the literature. Moreover, the achromatic broadband focusing property of the MMZP is independent with the polarization state of the incident light. This approach broadens degrees of freedom in micro-nano optical design, and is expected to find applications in multifunctional focusing devices and polarization imaging.

## 1. Introduction

To make optical components lightweight and multifunctional has always been one of the goals pursued in optics. Fresnel zone plate (FZP) is a typical representative [1,2,3,4,5,6,7,8]. In particular, developed on the basis of FZP, the multi-level diffraction lens (MDL) has achieved an achromatic imaging function range from visible to long-wave infrared bands via a globally optimized numerical iterative algorithm [9,10]. However, it can not modulate the polarization state of incident light while focusing with wide spectrum achromatic [11,12,13].

It is worth noting that the proposal of metasurface opens a new field of vision in the multi-functional design of optical components [14,15,16,17]. The free manipulation of the amplitude, phase and polarization of light by the metasurface has completely broken the limitation of optical materials [18,19]. Recently, many research studies devoted to the polarization transformation based on metasurface have appeared. Some of these works are also considered multifunctional optical elements which provide polarization transformation and focusing simultaneously [20,21,22,23].

Gwanho Yoon et al. proposed a new type metasurface called metasurface zone plates (MZP), achieved focusing and polarization manipulation for a single-wavelength via replacing the typical FZP rings by metasurface [24]. At present, MZP can achieve single-wavelength polarization conversion while focusing on several discrete wavelengths by using metal or dielectric subwavelength nano-antennas [25]. In addition, the focusing efficiency of these MZPs is generally low, just around 10% [26,27,28,29,30], and the highest focusing efficiency reported so far is 17% [31]. Obviously, it is still a very challenging task to achieve efficient broadband achromatic focusing while ensuring the control of polarization states. In my opinion, the main reason why MZP is not efficient is that all these research works replace the rings via metasurface, which discards the powerful amplitude control of traditional diffraction elements.

In this research, a novel nested composite structured multifunctional metasurface zone plate (MMZP) is designed via the combination of the diffraction theory and the electromagnetic resonance effect, which formed by integrating metasurface on the surface of the MDL rings. Based on the global optimization mathematical iterative method, the height distribution of the MMZP is optimized to realize the highly efficient achromatic broadband focus. Furthermore, the polarization state of incident light is accurately regulated by scanning and iterating the dimension parameters of the composite structure. This combination broadens degrees of freedom in micro-nano optical design, and is expected to find applications in multifunctional focusing devices, polarization imaging, and other fields [32,33,34].

## 2. Methods

**Figure 1:**
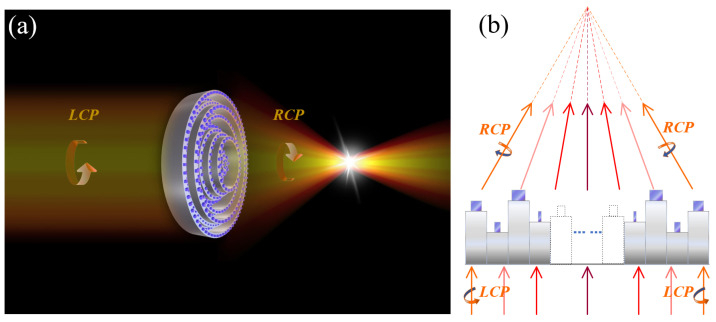
Schematic diagram of the MMZP designed for broadband focusing and polarization manipulation. The colors of the beams represent the different wavelengths. The arrow represents the spin state of the beam. (**a**) Three-dimensional diagram. (**b**) Sectional view.

The MMZP consists of several rings with fixed width and varying heights, each with a number of nanofins (usually called meta-atoms) above it. The high focusing efficiency over broadband wavelengths is achieved by selecting the multiple height levels dictated by the nonlinear optimization methodology. The polarization state of light can be manipulated, benefitting from the advantage of extreme form birefringence of metasurface. Herein, employing the diffraction theory and strong electromagnetic resonances simultaneously, broadband focusing and polarization-modulation are achieved. A left circularly polarized (LCP) light can be trasformed as a right circularly polarized (RCP) light. Figure 1a shows a schematic diagram for the MMZP.

To design an MMZP, the multi-level diffraction lens should be modeled first. After that, the phase profiles of each ring can be generated by changing the geometry parameters (length and width) of the nanofins. Then, the appropriate electromagnetic response produces the desired optical characteristics by the combination of MDL and metasurface as a novel hybrid design.

### 2.1. Design of Broadband MDL

The MDL design aims at high focusing efficiency over all wavelengths interested using a direct binary search (DBS) algorithm [35]. In essence, the design of MDL is a process of inversely solving the height distribution on the premise of knowing the desired optical field distribution of the imaging plane. For the broadband wavelengths, the diffracted field at the imaging plane is given by the Fresnel transformation [36]:

(1) $$U\left(x^{\prime },y^{\prime };\lambda \right)=\frac{e^{ikd}}{i\lambda d}\;\int \int \;g_{illum}\left(x,y;\lambda \right)T\left(x,y;\lambda \right)e^{i\frac{k}{2d}\left[{\left(x^{\prime }-x\right)}^{2}+{\left(y^{\prime }-y\right)}^{2}\right]}dxdy$$

where x′ and y′ are the coordinates of the image plane. *x* and *y* are the coordinates of the MDL plane. *λ* is the wavelength, and $k=\frac{2\pi }{\lambda }$ is the wave number. *d* is the propagation distance. $g_{illum}=1$ when the unit amplitude illumination wave is on-axis.

The corresponding transmission function of the MDL is

(2) $$T\left(x,y;\lambda \right)=e^{i\phi \left(x,y;\lambda \right)}=e^{ik\Delta h(n-1)}$$

where $\Delta h=\frac{h_{max}}{N_{levels}}$ is the height perturbation; $h_{max}$ is the maximum height of the profile; $N_{levels}$ is the total number of quanitization levels. Meanwhile, the *x* and *y* coordinates determine the ring width of MDL [36].

When designing the broadband MDL, the weight factor (noted as $\omega_{i}$) is introduced into the above model. Moreover, the continuous band is separated into *N* parts of operating wavelengths (where *N* is the given positive integer). At this point, the maximal average focusing efficiency of the broadband can be found by adjusting (increasing or decreasing operations for example) the height of each ring, while the width of each ring is fixed.

Obviously, the design problem of MDL has been transformed into a mathematical optimization problem. The numerical iteration algorithm is implemented to solve this nonlinear optimization problem. Given an initial height distribution of MDL, positive or negative height perturbation (Δh) is applied to each groove until the iteration termination condition is satisfied. Here, the iteration stop condition is defined as the figure-of-merit (FOM), which is coupled with the average focusing efficiency. The FOM is defined by [36]

(3) $$FOM=\frac{\sum_{i=2}^{N}\omega_{i}\mu_{i}}{N}-10\times \frac{\sum_{i=2}^{N}\omega_{i}\epsilon_{i}}{N}$$

where $\omega_{i}$ is the weight factor to balance contributions from different wavelength. *N* is the total number of the wavelengths. $\mu_{i}$ is called the efficiency, and $\epsilon_{i}$ is the normalized absolute difference, which can be expressed by the following two equations [36]:

(4) $$\mu_{i}=\frac{\;\int \int \;I_{i}\left(x^{\prime },y^{\prime }\right)F_{i}\left(x^{\prime },y^{\prime }\right)dx^{\prime }dy^{\prime }}{\;\int \int \;I_{i}\left(x^{\prime },y^{\prime }\right)dx^{\prime }dy^{\prime }}$$

(5) $$\epsilon_{i}=\frac{\;\int \int \;\left|normalize\left(I_{i}\left(x^{\prime },y^{\prime }\right)\right)-F_{i}\left(x^{\prime },y^{\prime }\right)\right|dx^{\prime }dy^{\prime }}{\;\int \int \;dx^{\prime }dy^{\prime }}$$

Here, $I_{i}\left(x^{\prime },y^{\prime }\right)={\left|U(x^{\prime },y^{\prime };\lambda )\right|}^{2}$ is the intensity at the image plane of the *i*-th wavelength. As the first-order approximation of a focusing point-spread-function (PSF), the objective function ($F_{i}\left(x^{\prime },y^{\prime }\right)$) is defined as a Gaussian function centered at $\left(\frac{x_{min}^{\prime }+x_{max}^{\prime }}{2},\frac{y_{min}^{\prime }+y_{max}^{\prime }}{2}\right)$ with full-width-at-half-maximum (FWHM) $W_{i}$ determined by the far-field diffraction limit [36,37]:

(6) $$F_{i}\left(x^{\prime },y^{\prime }\right)=\exp\left\{-\frac{{\left(x^{\prime }-\frac{x_{min}^{\prime }+x_{max}^{\prime }}{2}\right)}^{2}+{\left(y^{\prime }-\frac{y_{min}^{\prime }+y_{max}^{\prime }}{2}\right)}^{2}}{{\left(\frac{W_{i}}{2}\right)}^{2}}\right\}$$

(7) $$W_{i}=\frac{\lambda_{i}}{2NA}$$

(8) $$NA=\sin\left[\tan^{-1}\left(\frac{D/2}{f}\right)\right]$$

where $x_{min}^{\prime }$, $x_{max}^{\prime }$, $y_{min}^{\prime }$ and $y_{max}^{\prime }$ delimit the integration range from the leftmost to the rightmost of the MDL design. $\lambda_{i}$ is the *i*-th incident wavelength. NA is the numerical aperture. *D* is the diameter of the MDL and *f* is the designed focal length.

Note that the efficiency $\mu_{i}$ is defined proportionally to the focusing efficiency η, which is used as the power ratio of the focal spot (with a radius of just three times the FWHM spot size) to the total incident optical power. This means that the termination of the iterative calculation is conditional on maximizing the focusing efficiency. When the expect parameters (focal length, element aperture, ring width, and the material refractive index) are determined, the optimized height of each ring can be evaluated.

### 2.2. Design of the MMZP

To realize the polarization manipulation while maintaining broadband focusing, dielectric metasurfaces are introduced for the transmission phase modulation based on the MDL design. Metasurface is often used to generate new optical elements according to the geometric Pancharatnam-Berry (P-B) phase and dynamic phase. The P-B phase metasurface change the orientation angle of the nanofins to realze the dependent phase of transmitted or reflected light. For the dynamic phase metasurface, the phase modulation can be chieved by changing the geometry of the nanofin. Due to the interplay of the P-B phase and dynamic phase, dielectric metasurfaces can generate arbitrary polarization states, allowing light manipulation in the vectorial regime. In addition, the physical mechanism of the broadband focusing is based on the diffraction theory. It can be seen that the polarization characteristics and broadband focusing characteristics are independent of each other in our design.

When a birefringence nanofin is illuminated by a linear polarization light, the relation between the input ($E^{i}$) and the output ($E^{o}$), electric fields can be expressed as follows [38]:

(9) $$\left[\begin{array}{c} E_{x}^{o}\\ E_{y}^{o} \end{array}\right]=R\left(-\theta \right)\left[\begin{array}{cc} e^{i\phi_{x}} & 0\\ 0 & e^{i\phi_{y}} \end{array}\right]R\left(\theta \right)\left[\begin{array}{c} E_{x}^{i}\\ E_{y}^{i} \end{array}\right]$$

where $E_{x}^{i}$ and $E_{y}^{i}$ are the input electric field components of the *x* and *y* direction, and $E_{x}^{o}$ and $E_{y}^{o}$ are the output electric field components of the *x* and *y* direction. θ is the orientation angle of the anisotropic meta-atoms. Denote $\phi_{x}$ and $\phi_{y}$ as phase delays of the meta-atoms for *x*-linearly polarized (XLP) and *y*-linearly polarized (YLP) light. As we know, a linearly polarized (LP) optical wave can be viewed as a linear superposition of a LCP and a RCP optical wave. By selecting a group of suitable parameters of the nanofin, the relation between the modulation phase and the anisotropic meta-atoms’ properties is given by the formula [38]:

(10) $$\left\{\begin{array}{c} \left|\phi_{x}-\phi_{y}\right|=\pi \\ \phi^{+}\left(x,y\right)=\phi_{x}+2\theta \\ \phi^{-}\left(x,y\right)=\phi_{y}-2\theta \end{array}\right.$$

where $\phi^{+}\left(x,y\right)$ and $\phi^{-}\left(x,y\right)$ are the modulation phase of two arbitrary orthogonal states of polarization, which are based on $\phi_{x}$ and 2θ. The geometrical size and the refractive index of the nano-fin determin the dynamic phase $\phi_{x}$. The PB phase 2θ is related to the orientation angle.

According to this equation, when a series of meta-atoms with the phase $\left(\phi_{x},\phi_{y}\right)$ satisfy Equation (10), dielectric metasurfaces can achieve complete phase coverage to cover the whole Poincaré sphere in the design wavelength [24]. In our research, the LCP optical wave will be transformed into the right circularly polarization state after passing through the nanofin when the phase difference between $\phi_{x}$ and $\phi_{y}$ is π.

The phase difference created by the waveguide effect is described as $\frac{2\pi }{\lambda }n_{eff}H$, where $n_{eff}$ and *H* are the effective index and height of the unit cell [31]. Here, *H* is limited by the height of each ring in MDL. Then, the value of $n_{eff}$ can be changed by adjusting the geometrical size of the nanostructure when the material is identified. The phase difference can be numerically analyzed by the Finite-Difference Time-Domain (FDTD) solver (Lumerical Solutions, Inc., Vancouver, BC, Canada). Different from the classical MZP design, the ring width and ring height of MDL limit the period and height of the unit cell. Besides, the nanostructure parameters of each ring need to be optimized separately. Finally, the characteristics of broadband focusing and polarization regulation can be achieved simultaneously after combining the nano-fins on each ring, which maintains the same height as the MDL.

## 3. Results and Discussion

### 3.1. Broadband Focusing Characteristic of MDL

**Figure 2:**
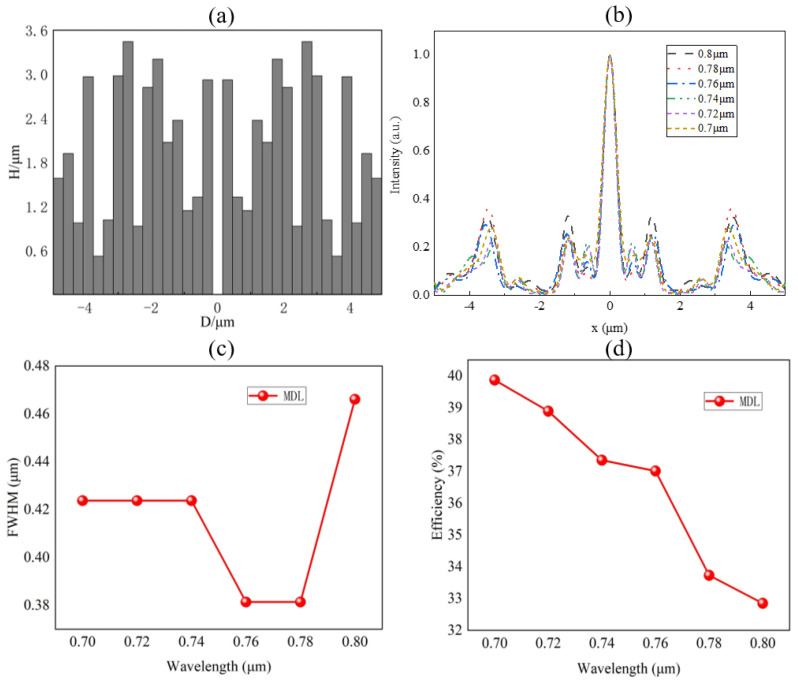
Focusing characteristic of the MDL. (**a**) Optimized height distribution. (**b**) Normalized intensity distribution along transversal axis (*x* axis). (**c**) FWHM and (**d**) Focusing efficiency at corresponding focal plane as a function of wavelength.

The MDL is designed for the wavelength range from 0.7 μm to 0.8 μm, which is a typical photoelectric detection band. The diameter is set as 9.9 μm, considering the computational load of numerical simulation. The width of each ring is fixed as 0.3 μm, which limits the period of the meta-atom for the polarization regulation in next step. Considering manufacturability, the material of the MDL is selected as SiO_2_ thin film. *NA* is 0.9 to get a good focusing effect. The iterative algorithm mentioned earlier is implemented programmatically. After 122 times of iterations, the optimized height distribution of the MDL is shown in Figure 2a. The maximum height is 3.5 μm, which provides a degree of freedom for phase control design. The height distribution is input into the FDTD commercial software to simulate optical field. Perfectly matched layers (PML) are applied at x and y directions and the propagation direction *z*.

Figure 2b shows the intensity distributions at the *x*–*z* plane. Focusing is accompanied with a sidelobe, which is also a common phenomenon for the diffraction elements. The FWHM as function of wavelength is plotted in Figure 2c. It is noted that all wavelengths achieve sub-wavelength focusing. The focusing efficiency is shown in Figure 2d. The average focusing efficiency in the near infrared band is above 33%, which is nearly 40% at 0.7 μm wavelength.

### 3.2. Hybrid Designed Results of the MMZP

**Figure 3:**
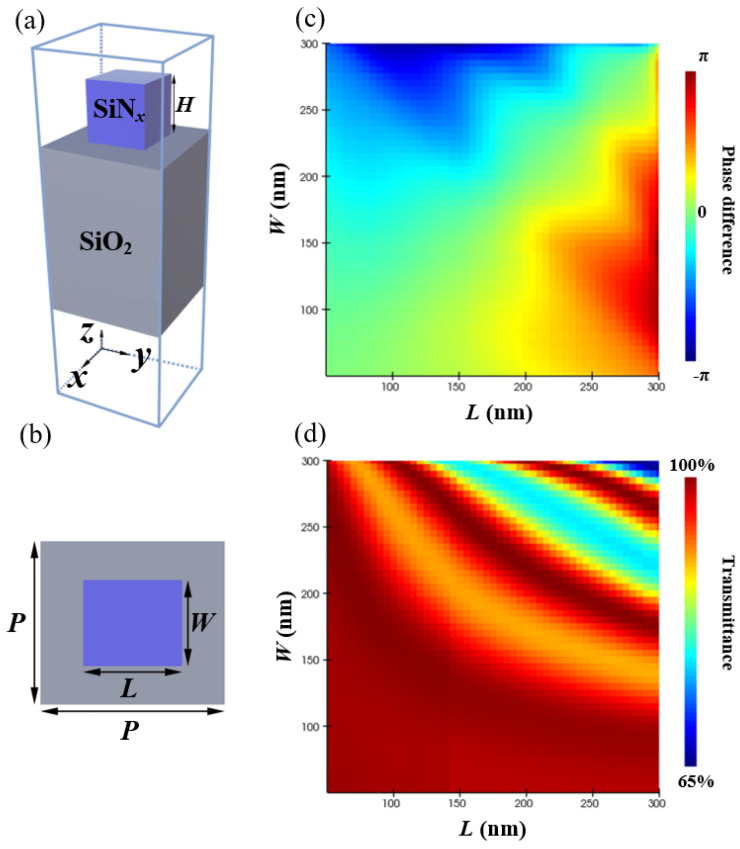
(**a**) Side-view and (**b**) top-view of the the unit cell: the period of a cell is *P*, and the length, width, and height are *L*, *W*, and *H*, respectively. (**c**) Simulated phase difference and (**d**) Simulated transmittance as a function of the nanofin size for the incident light of 0.7 μm.

To realize the phase difference of π between *x*-polarized and *y*-polarized light incident, the unit cell of the metasurface zone plate should be designed rigorously. The unit cell consists of a ${\mathrm{SiN}}_{x}$ meta-atom embedded on the SiO_2_ cell of the MDL ring as shown in Figure 3a,b. The high refractive index helps to reduce the aspect ratio of the structure. Si is not selected because the refractive index difference between upper and lower materials is too large, which will produce strong interference effect to interfere with broadband focusing [39]. The SiO_2_ and ${\mathrm{SiN}}_{x}$ used here have refractive index of 1.5 and 2.1, which can be prepared by plasma enhanced chemical vapor deposition (PECVD) [40].

**Figure 4:**
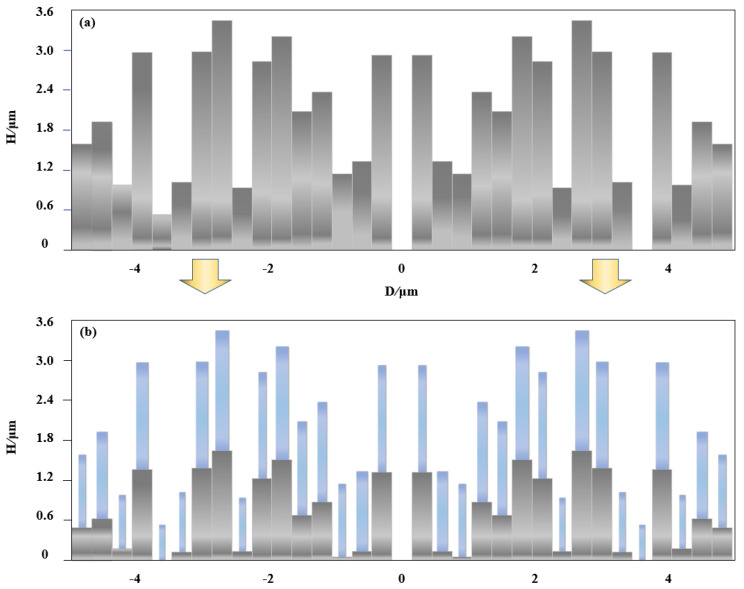
Height distribution of (**a**) MDL and (**b**) MMZP.

**Figure 5:**
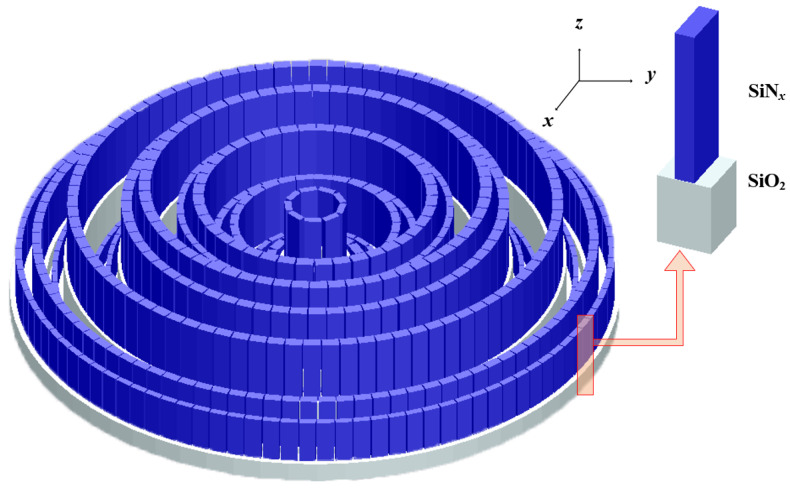
The three-dimensional drawing of designed MMZP.

The FDTD method is implemented to obtain the phase difference range and the transmission coefficients as shown in Figure 3c,d. The incident wavelength is plane wave with an *x* or *y* polarization and propagates along +z direction. Periodic boundary conditions (PBC) are applied at *y* directions and perfectly matched layers (PML) at the directions *x* and *z*. Here, the height of the meta-atom is limited by the height distribution of the MDL, and the refractive index of the material is also determined. The length (*L*) and width (*W*) swepting from 50 to 300 nm of the nanofin can be changed to obtain the suitable value for the phase difference of $\left|\phi_{x}-\phi_{y}\right|$. As can been see from the results, the corresponding phase difference span from −π to π, and meanwhile the transmissions can reach up to more than 90%. For example, for the first ring, the height of SiO_2_ is set as 0.5 μm, the height of ${\mathrm{SiN}}_{x}$ is set as 1.1 μm. We select L=290 nm and W=110 nm, then the phase difference can be *π*, which is our expected value. This calculation should be performed 17 times to obtain the required structural parameters. Figure 4 shows the height distribution of MDL and MMZP. Both are 9.9 μm in diameter. They maintain the same height distribution. The difference is that MMZP is composed of nested composite structures. The meta-atom building blocks are arranged into periodic arrays, while the three-dimensional drawing of designed MMZP is shown in Figure 5.

### 3.3. Broadband Focusing and Polariztion Manipulation of the MMZP

**Figure 6:**
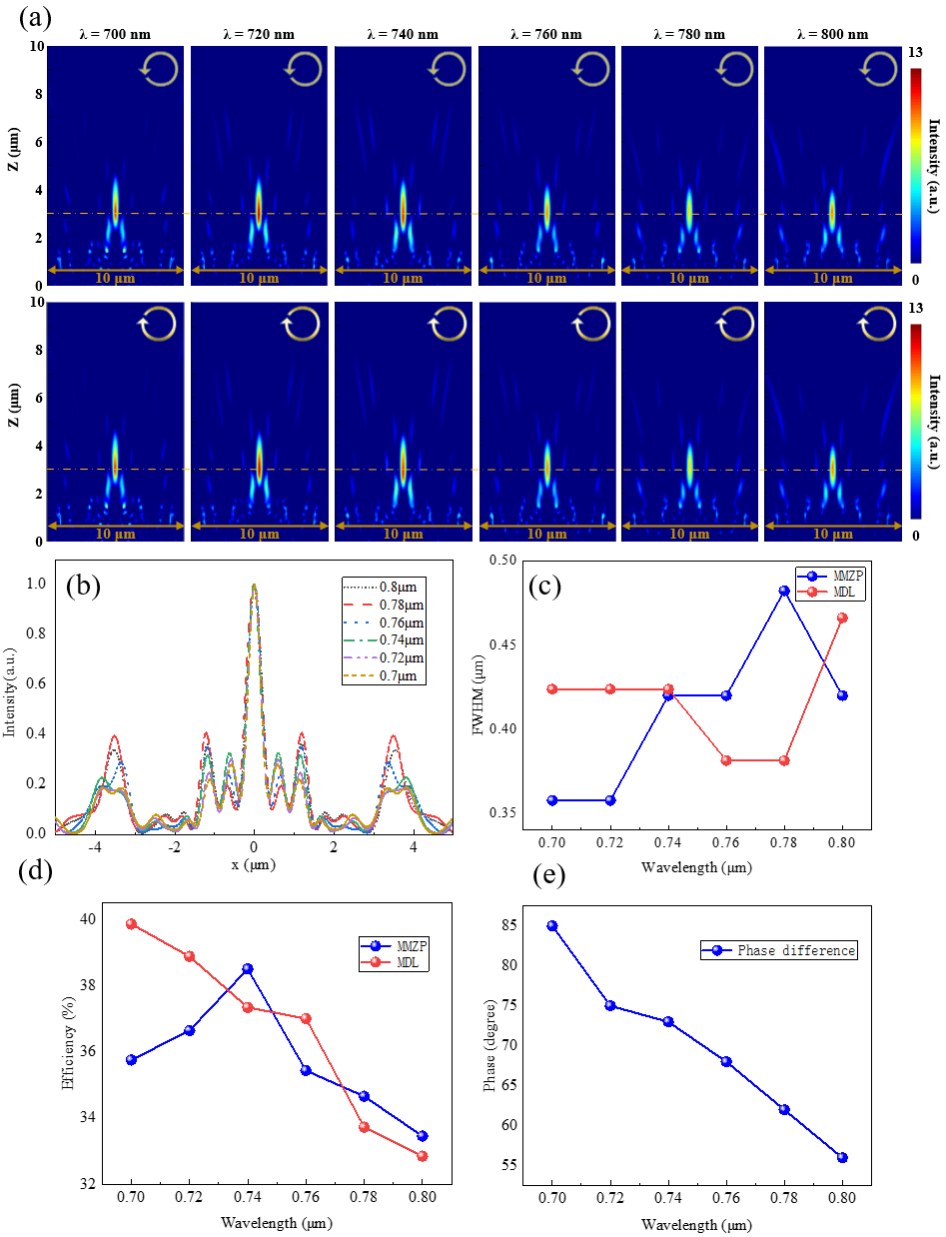
Focusing characteristic and polarization manipulation of the MMZP. (**a**) Numerical intensity profiles along axial planes at various incident wavelengths for LCP (top row) and RCP (bottom row) incident light. (**b**) Normalized intensity distribution of the MMZP along transversal axis (*x* axis). (**c**) FWHM and (**d**) Focusing efficiency at corresponding focal plane as a function of wavelength. (**e**) Simulated phase difference of various incident wavelengths for LCP light.

In order to verify whether the hybrid design can keep the broadband focusing characteristic and realize the polarization manipulation at the designed wavelength simultaneously, thirty-three kinds of structures are used as meta-atoms to simulate the behavior of the MMZP incident by the circularly polarized light. PML are applied at *x* or *y* directions and the propagation direction *z*. The simulated results can be seen in Figure 6.

Figure 6a shows the intensity distribution of the cross section plane. The focal length maintains almost the same while incident light varies, verifying the realization of a near-infrared achromatic broadband focusing feature. Due to the structural characteristics of rotational symmetry, LCP and RCP light have almost the same light field distribution. Figure 6b shows the intensity distributions at the *x*–*z* plane. The FWHM and the focusing efficiency is shown in Figure 6c,d. All FWHM are nearly half of the corresponding incident wavelength. The focusing efficiency decreased by about 3% compared to MDL, which is due to the reduced duty cycle of the structure resulting in the decrease of energy utilization. Figure 6e shows the results of polarization manipulation. The incident LCP light is set as the superposition of a XLP and YLP light, which has a phase difference of −90°. For the design wavelength of 0.7 μm, the phase difference is close to 90°, which meets our expectation. It means the incident light can be converted from left circularly polarized light to right circularly polarized light after passing through the MMZP. It can be seen that the hybrid design can realize the polarization manipulation while keeping the broadband achromatic in the near-IR.

### 3.4. Polarization-Insensitive Feature of the MMZP

**Figure 7:**
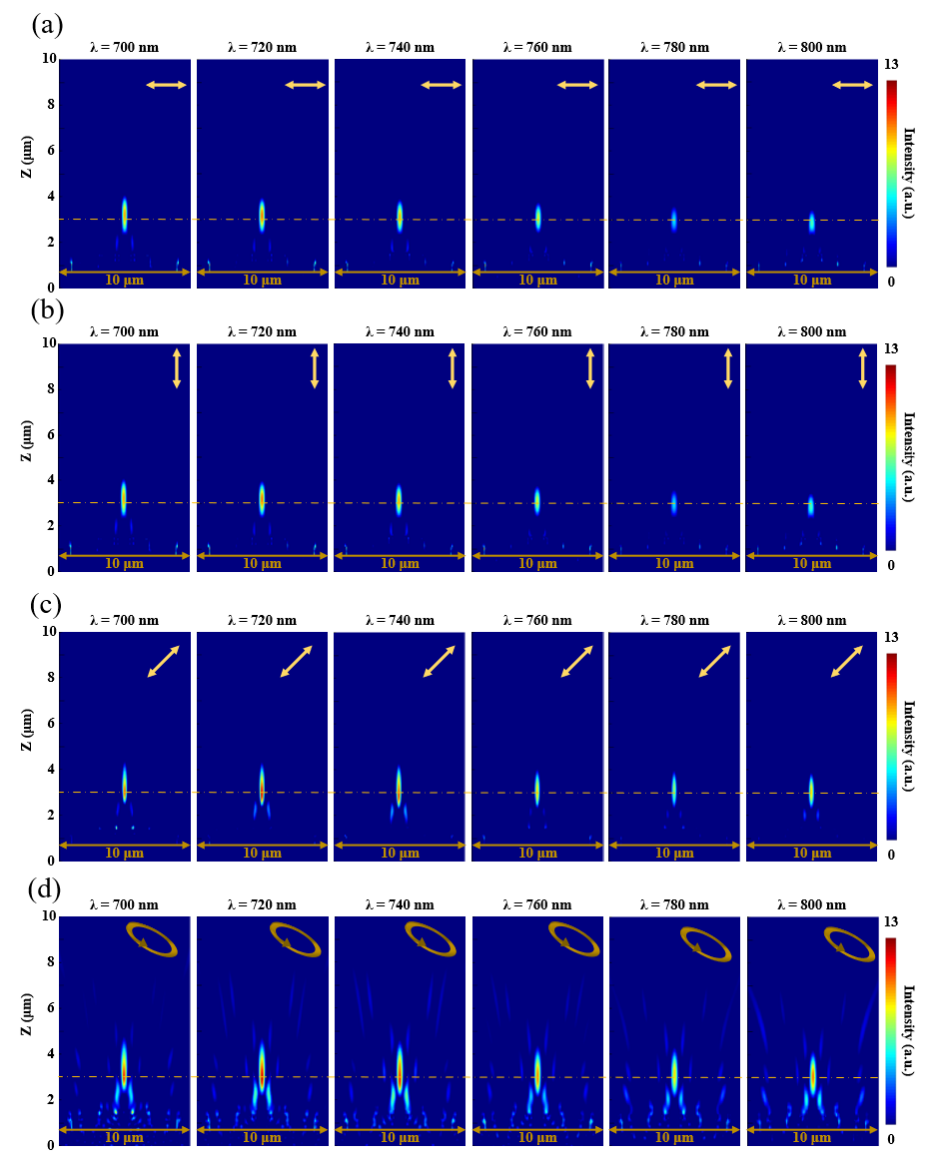
Numerical intensity profiles along axial planes at various incident wavelengths for (**a**) XLP, (**b**) YLP, (**c**) $45^{\circ }$ linearly polarized ($45^{\circ }$-LP) and (**d**) elliptically polarized (EP) incident light.

The MMZP exhibits insensitivity to the polarization of incident light. Figure 7a,b show the normalized intensity distributions at the *x*–*z* plane under the incidence of XLP and YLP light, respectively. Figure 7c,d show the normalized intensity distributions at the *x*–*z* plane under the incidence of an arbitrary linearly polarized light ($45^{\circ }$) and the elliptically polarized incident light. The MMZP maintains the broadband achromatic property when the incident light is in different polarization states.

**Figure 8:**
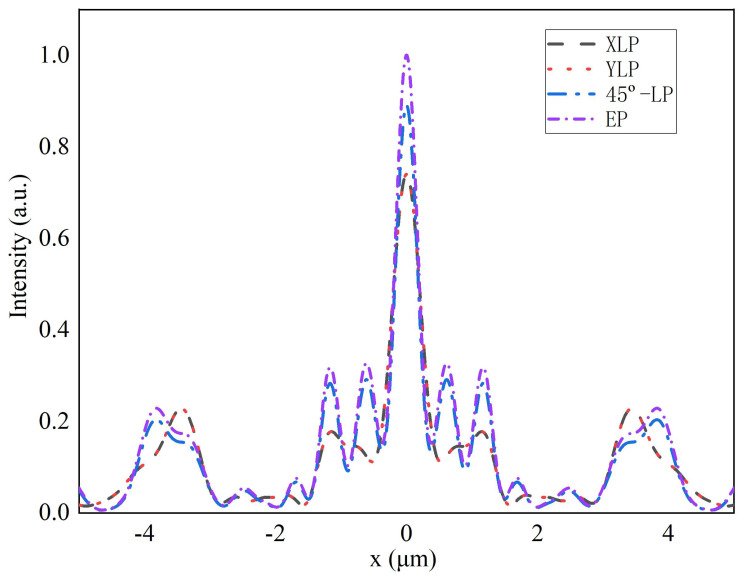
Numerical intensity profiles along axial planes at various incident wavelengths for Intensity distributions of the corresponding focal plane.

Figure 8 shows the FWHM of 0.74 μm wavelength with the different polarization states. After passing through the MMZP, the elliptically polarized incident light has the strongest light intensity, followed by the linearly polarized light (45°). The XLP and YLP light has the weakest light intensity. The results indicate the polarization-insensitive feature of MMZP.

It can be seen that the LCP light can be converted into RCP light while achieving efficient achromatic broadband focusing under normal incidence. In fact, by adjusting the geometry of the composite structure, the light wave of any polarization state can be obtained by this method [24]. Based on this, the application of MMZP in polarization imaging is the next step to be carried out. Besides, the refractive index of the coating changes with the temperature. Therefore, temperature certainly has an effect on focusing and even polarization modulation. Discussion of the relevance of the temperature dependence of the coating is expected to be carried out in the further research.

## 4. Conclusions

In summary, unlike typical MZPs, which use metasurface instead of diffraction rings, the novel nested composite structured MMZP was successfully modeled by integrating metasurface on the surface of the MDL rings. Based on the global optimization mathematical iterative method, the height distribution of the MMZP is optimized to realize the highly efficient achromatic broadband focusing. The focal length maintains almost the same while incident wavelength varying from 0.7 to 0.8 μm, indicating the realization of a broadband achromatic converging property in near-IR region. The focusing efficiency reaches above 35%, which is more than twice as much as reported in the published results. Furthermore, the polarization state of incident light is accurately regulated by scanning and iterating the dimension parameters of the composite structure. These results indicate that the MMZP has promising practical application prospects in multifunctional focusing devices and polarization imaging.

## Data Availability

Data are contained within the article. The data presented in this study are available in Section 2 (Methods) and Section 3 (Results and Discussion).
